# Overview of Poultry Management as a Key Factor for Solving Food and Nutritional Security with a Special Focus on Chicken Breeding in East African Countries

**DOI:** 10.3390/biology10080810

**Published:** 2021-08-20

**Authors:** Lenox Omondi Pius, Péter Strausz, Szilvia Kusza

**Affiliations:** 1 Animal Breeding and Genetics Resource Section, Tanzania Livestock Research Institute (TALIRI), P.O. Box 834, Dodoma 41207, Tanzania; piuslenox@yahoo.com; 2 Department of Management and Organization, Institute of Management, Corvinus University of Budapest, 1093 Budapest, Hungary; peter.strausz@uni-corvinus.hu; 3 Centre for Agricultural Genomics and Biotechnology, University of Debrecen, 4032 Debrecen, Hungary

**Keywords:** challenges, East Africa, food and nutritional security, genetic improvement, indigenous chicken, production system, prospects

## Abstract

**Simple Summary:**

The poultry sector in most developing countries is largely based on traditional production systems, which are based on indigenous breeds. Beyond economical, nutritional, socio-cultural, and religious functions, the inherent adaptability of indigenous birds to diverse environmental conditions also provides a unique genetic resource critical for addressing the global challenges of food security in this world impacted by climatic change and human population growth. Nevertheless, until recently, the potential of the indigenous chicken remained largely untapped for no strong reason. In this review, we offer an overview of food and nutritional security with a special focus on chicken breeding in East Africa. We highlighted and combined confirming evidence of production performance, phenotypic variability, and genetic diversity of East African indigenous chicken using both morphological and molecular tools. Previous attempts to improve the productivity of indigenous chicken are highlighted, and possible future breeding plans and areas of immediate research are suggested. Considering how indigenous chicken strongly affects the livelihood of the majority of households, and since the poultry sector is likely to be strongly affected by climate change, we recommended that the prospects of chicken breeding in Africa should create a permanent balance between the competing needs of genetic improvement and the genetic diversity of indigenous chickens.

**Abstract:**

The focus of this review is to offer an overview of food and nutritional security, to identify associated constraints, and propose possible alternative solutions for improving the East African poultry sub-sector in the pursuit of food security, focusing on chicken breeding. To better understand the prospects of the poultry industry, we highlighted and combined confirming evidence of the phenotypic variability and genetic diversity of East African chicken genetic resources using both morphological and molecular tools, as well as performance traits. Furthermore, this work gives a detailed indication of what would be lost if indigenous chicken populations are left to suffer the ongoing massive genetic erosions due to various factors, not limited to indiscriminate crossbreeding. Previous and recent attempts to improve the productivity of indigenous chicken are highlighted, and possible future breeding plans and areas of immediate research are suggested as well. This review concludes that under the prevailing conditions, the village chicken production system appears to be the most imperious production system that needs to be extensively developed ; however, for the sustainability of the industry, the proposed improvement strategies should create a permanent balance between the competing needs of genetic improvement and the genetic diversity of the indigenous chicken genetic resource.

## 1. Introduction

Food security and supply are a significant concern facing the world today, with the most recent estimates revealing that 795 million people, nearly one out of ten people in the world, are affected by chronic undernutrition, with most severe cases of hunger, malnutrition, and health-related undernutrition in Africa [1]. According to the World Bank, the proportion of the people in Africa who live in extreme poverty and undernourishment has decreased considerably from about 57% in 1990 to about 41% in 2013 [2,3]. Nevertheless, until recently, the largest share of the global population of extremely poor and undernourished people, of more than all other regions combined, still live in Africa [1,2]. Considering the predicted increase in the human population in Africa, which is expected to double its current figure of 1.2 to more than 2 billion by 2050, food insecurity concerns may be worsened in the near future [2]. The projected demand for protein is of particular interest in view that the world’s demand for animal-derived protein will double in the same period, imposing more threats of undernourishment in Sub-Saharan Africa (SSA) [4]. Furthermore, due to the health and socio-economic impacts of COVID-19, the nutritional status of the most vulnerable population groups is likely to deteriorate in the near future. Generally, the disturbing food insecurity concerns in Africa are partly caused by climate change effects, which in turn are influenced by the limited adaptive capacity of the agricultural system, which highly depends on rain-fed agriculture and poor policies [1,5]. Correspondingly, the deteriorating climate circumstances, such as droughts and floods, exacerbate the vulnerability of the agricultural system in the region [1]. The East African region is also among the regions most vulnerable to food insecurity issues in SSA, with some of the highest rates of undernutrition globally [6,7]. East Africa’s economies are heavily dependent on small-scale agriculture, with at least two-thirds of all food production in the region coming from smallholder farms of less than 2 hectares and with minimal livestock holdings [6,8]. Thus, the agricultural sector, which employs a high proportion of the population and over 90% of some rural populations, while contributing about 25-30% of the national income in most low-income countries, is left in the hands of poorly equipped smallholders [1]. Other roadblocks in attaining food security in the region include low technological capability, insignificant economic growth, increasing populations, unstable social and political environments, macroeconomic imbalances in trade, natural resource constraints, natural disasters, high weather variability and climate shocks, poor food distribution network within the member countries, and inadequate food trade between the member countries [5,9]. Each of these issues influences the others, making the food security situation in the region complex and hazardous [10]. Nevertheless, the region has a huge untapped potential to produce enough food for themselves and a surplus for trade if appropriate and functional food production measures are put in place [2,11]. In the meantime, efforts to sustain and enhance the productivity of smallholder farmers and agro-pastoralists in the face of climate change and other threats to regional food systems could have a positive effect on broader regional food security goals. Therefore, the present study offers an overview of food and nutritional security, discusses the existing challenges, examines poultry breeding and production systems, emphasizes the enormous role of the smallholder production system in maintaining food and nutrition security, highlights previous attempts for genetic improvement, and finally provides an alternative solution (prospect) to the problem of the food supply with special focus on chicken breeding in three East-African countries, namely Kenya, Uganda, and Tanzania.

## 2. Methodology

This research consists of a scoping review of the literature offering an overview of poultry management as a key factor of solving food and nutritional security in East Africa. East Africa is a diverse region that consists of Djibouti, Eritrea, Burundi, Ethiopia, Kenya, Tanzania, Uganda, Rwanda, Somalia, Sudan, and South Sudan. This review, however, de-limited the East Africa region to include three East African Community partner states, namely Kenya, Uganda, and Tanzania. For each country, we manage to examine the current situation of food and nutrition; we offer an overview of the poultry sector and its corresponding contribution to the economy of the respective countries, including their per capita consumption rates. With regards to breeds and breeds distribution, we managed to discuss the production, reproduction, and adaptation potential of different breeds, including evidence from their morphological and molecular characterization studies. Past, present, and future breeding plans are also discussed, and some recommendations that are likely to improve poultry breeding in East Africa in the long term are provided. A comprehensive search was conducted to identify eligible peer-reviewed academic articles and grey literature, including publications from international institutions, such as the World Bank, the Food and Agriculture Organization of the United Nations, and the USAID, which always play a significant role in formulating policies and interventions targeting to defeat anger within the agricultural sector in developing countries. Recent government official reports, such as budget speech, Livestock Sector Developmental Plans and policies, and data from the National Bureau of Statistics have also been included in this review to address the most recent developments that have not been reflected in the academic press. Publications were identified through searches of academic databases, such as the web of science, Google Scholar, and the Wiley Online Database. The search strategy involved a combination of the following keywords: East Africa, food and nutritional security, challenges, IC, production system, prospects, genetic improvement. The remaining relevant publications were extracted from the reference lists of the reviewed documents, Directory of Open Access Journals, as well as from the knowledge of co-authors.

## 3. Food Insecurity and Nutritional Status in the EAC Region

### 3.1. Food Security and Nutrition Profile Overview in Kenya

Agriculture is the second largest contributor to Kenya’s economy after the service sector, contributing about 33% to the GDP and about 75% of Kenyans earn all or part of their income from the sector [10,11,12,13]. Among many other obstacles, agricultural productivity is constrained by agro-ecological problems, including recurrent crises, such as droughts, floods, and intemperate weather, which largely contribute to the high malnutrition levels in the country [13]. It is estimated that 80% of the total agricultural land is arid or semi-arid, and only the remaining 20% (5.8 million ha) of Kenyan land is suitable for farming [10]. Furthermore, 15–20% of Kenya’s land area regarded as suitable for farming is not utilized efficiently [10]. With these constraints, Kenya continues to face severe food insecurity, and about 3.4 million people suffered from acute food insecurity in 2017 [14]. In the 2020 Global Hunger Index, Kenya ranks 86th out of 117 qualifying countries with enough data to calculate Global Hunger Index [15]. With a slightly higher proportion (23%) of the undernourished population and a score of 25.2 in the 2019 Global Hunger Index, Kenya suffers from a level of hunger that is termed serious [15]. Malnutrition is a public concern and is the single most important contributor to child mortality, which stood at 30.6% of every 1000 births in 2018, down from 33.3% in 2015. Malnutrition is mainly due to inadequate food intake and disease, with the underlying factors being household food insecurity, poor childcare practices, and inadequate sanitation and health care services, among others [16,17]. Nevertheless, Kenya has made substantive progress in reducing the prevalence of stunting nationally, falling from 35% to 26%, and the wasting rate from 7.0% to 4.0% by 2008 [14]. According to Table 1, between 2015 and 2019, the proportion of wasting and stunting in children under five years stood at 4.9% and 31.3%, respectively [15].

### 3.2. Food Security and Nutrition Profile Overview in Uganda

Uganda is a low-income country with agriculture as the mainstay of its economy. Agriculture contributes about 24.6% to GDP, provides half of the export earnings, and employs 71% of the population [18]. According to the estimates made by the World Bank, 41.7% of the Ugandan population lives under the poverty line, only 25% of the population is food insecure, and the remaining 75% gets at least one basal meal a day [19]. Agricultural productivity is constrained by a variety of institutional and economic bottlenecks, as well as agro-ecological constraints, with estimates that 44% of the total agricultural land of the country is semi-arid [18]. With the Global Hunger Index score of 30.6, Uganda suffers from a level of hunger that is termed serious (Uganda was recently ranked 106th out of 177 out of qualifying countries [15]. Data registered from 2015 to 2019 (Table 1) revealed that the wasting and stunting rate in children under five years stood at 3.5% and 28.9%, respectively, i.e., almost one out of three children under 5 years in Uganda is stunted [15].

### 3.3. Food Security and Nutrition Profile Overview in Tanzania

In Tanzania, the agricultural sector (livestock-inclusive) forms the mainstay of the country’s economy in the sense that it generates 25% of the GDP, contributes 30% of export earnings, employs over 75% of the country’s total labor force, while offers livelihood support to over 80% of the population [20,21]. It is estimated that approximately 46% of Tanzania’s total landmass is suitable for agricultural production. According to the preliminary food crops forecast report 2015/2016, the food self-sufficiency ratio (SSR) over the period of four years from 2012 to 2015 was estimated to be over 100% (123), implying surplus food production at the national level. The above observation indicated the possibility that the country can be a net exporter of food if appropriate and functional food production strategies are put in place. Despite significant improvements in recent years, Tanzania has a high level of malnutrition among children and women. Tanzania has made a significant improvement in wasting and underweight indicators; however, stunting has remained persistently higher and varied between regions [22]. In the past 25 years (1991–2016), the burden of stunting in Tanzania has declined by 30% [23], and between 2014 and 2018, stunting was reduced from 34.7% to 31.8% [22]. Despite this progress, it is estimated that more than 2,700,000 children (one in every three children) under five years of age are stunted in 2019 [22]. Moreover, the prevalence of malnutrition among children under the age of five years has been decreasing in the last two decades from 31.1% in 2000 to 25% in 2019, while wasting and stunting in children are at 3.5% and 31.8%, respectively [15]. In the 2020 Global Hunger Index, Tanzania scored 25.0 and ranked 89th out of the 107 qualifying countries with sufficient data to calculate the Global Hunger Index (Table 1). Thus, efforts are needed to increase the pace of reducing stunting, especially among children in rural areas; these important determinants should be addressed through effective and tailored nutrition-sensitive and specific interventions using multisectoral approaches [23].

## 4. The East-African Poultry Sector—An Overview 

The East Africa community is an agro-based economy region with agriculture as one of the essential sectors that offer employment opportunities to the majority of the population for their livelihood. The livestock sector, including the poultry sector, is a part of the agricultural sector that contributes tremendously (20–30%) to national GDP, as well as to the social-economic development of the region. The poultry sector in the Eastern African countries is not well developed, although over the past couple of decades, it has grown from a backyard poultry keeping operation to a more commercial-oriented system [24]. The increasing human population, urbanization, and increasing income have been strong drivers of the growth. In general, the current state of the poultry sector does not strongly differ from those of almost all other Eastern African countries; however, Kenya’s poultry sector is recognized as the most mature [24]. Despite efforts to develop a commercial intensive poultry production system in East Africa, the majority of poultry are still kept by smallholders in less intensive systems, largely based on indigenous chicken (IC) breeds [25]. The empirical literature suggests that East African states suffer seriously from several challenges that impede the effective development of the poultry sector in these partner states. In summary, the African poultry sector in general is commonly affected by interactive factors, not limited to low genetic potential of the indigenous poultry flock, unreliable supply of day-old chicks, inconsistent supply of quality feeds, high mortalities caused by high disease prevalence, poor access to inputs and services, limited credit facilities, poor marketing infrastructure and fragmented supply chain, malpractice and poor quality of chicks caused by the overwhelming demand for hatcheries, low investment capacity and inefficient production methods, weak transportation infrastructure, lack of sophisticated technology, unfavorable policies, unhygienic slaughtering and storing facilities, inadequate quality control to meet market and phytosanitary standards, inadequate value addition capacity (ineffective packaging, grading, branding and certification, poor access to financial services, poor supply of utilities (e.g., electricity)), shrinking government agricultural budget [24,25]. Despite the relative growth of the commercial poultry sector in the recent past, the village chicken production systems remain the most important subsector and a better alternative to the commercial sector in all East-African countries. The advantages of these systems are the low levels of inputs that they require [25,26,27] and the unique products they produce, which provide essential sources of livelihood, food security, and nutrition support to millions of resource-poor farmers. It is estimated that rural poultry represents up to 80% of the total poultry population in tropical countries, supplying nearly 100% of poultry meat and eggs consumed in rural areas and about 20% in urban areas [24,25]. Even if chicken’s meat is consumed infrequently in rural areas, the provision of eggs throughout the year can help to mitigate the effects of seasonal food unavailability in many ways [25,26]. Rural poultry provides not only the best quality protein from meat and eggs but also essential vitamins and minerals that are badly needed for the wellbeing of millions of undernourished people, especially pregnant women and children who often live in poverty [25,26,27,28,29]. Apart from being a direct source of food for poor rural households, sales from poultry products often generate substantial income and servings, particularly for women, thus enhancing the capacity of the family to cope with shocks and reducing economic vulnerability [25,28]. Even though the contribution of poultry-keeping to household cash income is deemed to be difficult to assess, it was estimated that an average flock of three hens and two cocks in Central Tanzania provides an additional income equivalent to 10% of the average annual income, i.e., USD 38 [25,30]. Beyond the economic and nutritional importance, socio-cultural and religious functions of village poultry production for smallholder livelihoods are also widely recognized [25,28]. Nevertheless, the potential of the indigenous poultry industry remains largely untapped, as evidenced by their extremely low productivity and small production scale. The majority of households keep 5–15 adult chickens, with only 3% of the households raising more than 40 birds [25,28]. However, the demand for IC meat and eggs remains high, mainly due to the preferred taste of the chickens and the generally trusted methods of raising the birds, which consequently command a premium price [27,31]. 

## 5. Importance of the Poultry Sector to the Economy; Meat and Egg per Capita Consumption in East-Africa 

In Kenya, livestock production is an integral part of the agricultural sector, contributing 4.4% of the country’s GDP and 30% of the agricultural contribution to the country’s GDP [32]. The sector employs 50% of the agricultural labor force and generates a significant number of jobs along the value chain [33]. According to recent statistics, the country’s animal population comprises 18.8 million cattle, 26.7 million goats, 18.9 million sheep, 3.2 million camels, 1.9 million donkeys, 0.5 million pigs, and 44.6 million poultry [33,34]. About 5.5 million households, close to half of the Kenyan households, keep poultry [33]. The Kenya poultry sector contributes more than 35,000 tons of meat in the market per year and about 1.6 billion eggs [35]. The per capita consumption of poultry meat and eggs is 0.6 kg and 45 eggs per year, respectively [33,36], while the per capita consumption of all kinds of meat is 15.6 kg of meat and 121 L for milk per year [33].

According to the recent statistics provided by reports issued by Uganda’s Ministry of Agriculture, Animal Industry and Fisheries, and the Uganda Bureau of Statistics, there were 14.5 million cattle, 16.1 million goats, 4.6 million sheep, 4.2 million pigs, and 48.9 million poultry in the country in 2019 [37]. The livestock sector accounts for 3.8% of the GDP in Uganda, and 58% of the households were registered to depend on livestock for their livelihood [37]. Out of the national poultry flock (48.9 million), 87.7% of the population are IC, which are largely reared in the free-ranging system [37]. It is estimated that 40% of all households in Uganda raise chickens, which contributes about 7–18% to their household income [37,38]. In the financial year 2019/2020, the poultry sector in Uganda experienced steady growth of about 11.4% compared to the 9.8% growth recorded in the previous year. According to the Ugandan Bureau of Statistics, this was the highest growth of the sector recorded so far in the last 10 years [37]. It was, however, worth noting that high growth was part of inspirational strategies made in the Second National Development Plan of 2015–2020 and the Agriculture Sector Strategic Plan of 2015–2020 of Uganda, which focus on beef and chicken sub-sectors as the priority commodities for commercial development in the future [38,39]. In 2018, nearly 0.93 billion eggs were produced in Uganda [Table 2]. Furthermore, it is estimated that nearly 65,000 tons of chicken meat are annually produced in Uganda [38]. The total supply of animal source foods in the country translates into a per capita consumption of about 14 kg of meat and 36 L of milk per year. The per capita consumption of chicken meat is 0.8 kg and 22 eggs per year [18].

Tanzania is regarded as the country in Africa with the second-largest livestock population after Ethiopia [40]. According to the Tanzania Livestock Sector Analysis (2016–2032), the livestock population is estimated to be 33.4 million cattle, 21.29 million goats, 5.65 million sheep, and 2.14 million pigs in 2020 [41]. The chicken population, which is the focus of this review, is estimated to be 83.28 million, of which 38.77 million are IC, and the remaining 44.51 million are exotic poultry [41]. Other species in the country include ducks, guinea pigs, turkeys, rabbits, camel, and water buffalo, which are considered less important to household income and food security as their numbers are fewer and held by fewer households [42]. Despite being ranked second in Africa in terms of cattle population, livestock-related activities contributed only 7.4% to Tanzania’s GDP in 2019, and the growth of the livestock sector at 5% is considered low [41]. Of the sector’s contribution to the country’s GDP, about 40% originates from beef, 30% from milk, and another 30% from poultry and small stock production [40]. According to the Ministry of Livestock and Fisheries, the poultry sector contributed nearly 81 thousand metric tons of meat to the market share, which was about 12% of the total annual meat production in 2019 [41]. It is estimated that in the last 5 years, nearly 3.6 billion eggs were produced annually (Table 3). The annual per capita consumption of poultry meat and egg is 0.7 kg poultry and 13 eggs per annum [41]. These levels are, however, extremely low when compared to the overall per capita consumption levels for Sub-Saharan Africa, which is approximately 147 eggs and 9.5 kg of poultry meat per annum [43]. The per capita consumption of all kinds of meat and milk in the country is 14 kg and 36 L per year [44]. Tanzania’s livestock sector (poultry sector inclusive) has, for a long time, been based on traditional livestock production practices as evidenced by a higher population of indigenous breeds, which have always dominated the national population. Nevertheless, the number of exotic chickens has been steadily increasing since 2013, while that of ICs has been slightly decreasing [40]. The slow growth of the livestock sector is mainly due to low investment, high mortality rates, low reproductive rates, and poor quality of the final products. The traditional sub-sector is also challenged with limited access to inputs and services, including improved genetic stock, extension services, financial services, and output markets [40,41].

## 6. Description of Chicken Breeds, Their Growth and Production Performance, Distribution, and Adaptation Potential in East Africa

Africa has a wide variety of IC that exhibit vast variability in their phenotypic appearance with no standard characteristics. Distinct phenotypic variations among African IC have been documented in a number of studies in Uganda [45], Kenya [46,47,48,49,50,51], Tanzania [52,53,54,55], Ethiopia [56,57,58], and many other countries. The variations among African IC discussed in this review mostly comprise body size and morphology, feather patterns and morphology, shank color, comb and wattle types, earlobe color, as well as productivity traits [59]. For the IC population characterized so far in the above-mentioned countries, three sub-populations (dwarf, medium-sized, and heavyweight) have been identified based on body size differences [46]. While the majority of the IC population exhibit normal plumage morphology and distribution, other unique features, such as naked-neck, frizzle, feathered shank and feet, muffs and beard, crest, and silkiness variants are also well represented within the population [47,51]. Furthermore, regarding plumage pigmentation, IC have more distinguished pigmentation, although the majority varies widely with black, brown, or red colors showing extensive and mottled colorations [46,50,51]. Irrespective of their plumage color, the majority of IC have red combs and wattles; however, a few birds have their wattle mottled-red with white and black spots [47,51]. The majority comb type is often single nevertheless, some exceptional appearances, such as strawberry, cushion, rose, buttercup pea, walnut, duplex, and crest, also exist [47,50,51]. Further, the shank and skin pigmentation varies in the range of yellow, white, black, green, and brown color [46,47,51]. While most of the birds have red eye lobes, other colors, such as white and molted red, also occur in a small proportion [47]. 

### 6.1. Production Performance of IC Ecotypes

Previous studies have also revealed the existence of considerable variation among the IC population concerning production performance (Table 4). The biological productivity of IC is very low and highly variable relative to commercial birds, as evidenced by low body weight, less egg production, high chick mortality rate, and many other unfavorable traits. Nevertheless, previous findings have indicated the existing potential of reducing these productivity gaps if feed, health, and strategic breeding programs are engaged [60,61,62]. The average age at sexual maturity in most IC ranges from 6 to 7 months under the free-ranging system, though these records have been reported to improve up to 5.5 months when better management practices were engaged [63]. Similarly, the average annual egg production of IC is around 45 eggs, although with improved management, egg production has been shown to improve up to a range of 120–160 eggs with some IC ecotypes [60,61,63]. According to Table 5, IC normally produces 3–4 clutches per year, with a range of 11–15 eggs/clutch. The incubation of eggs is always by natural brooding, and hatchability is usually very high (more than 75%); however, artificial incubation is still providing lower hatchability rates than natural brooding [60]. With regard to growth performance, the mature bodyweight for cocks has been reported to range from 1.2 to 3.2 kg and 0.7 to 2.1 kg for the hen [45,54,64]. However, with improved management, an increase of up to 25% in body weight can be achieved with some IC ecotypes [60,61,65]. 

### 6.2. Major Indigenous Chicken Genetic Groups in East Africa, Their Distribution and Adaptation Potential

**Figure 1:**
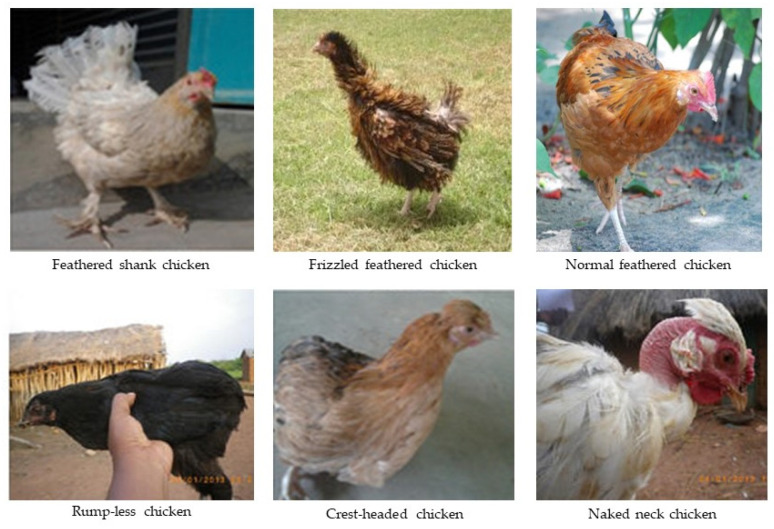
Unique attributes of indigenous chicken.

Most African IC populations have not been conclusively characterized into standard breeds until lately. The available IC genetic groups have been classified based on the phenotypic expression of their major genes or their geographical location [46,47,53]. For the indigenous African chicken population characterized so far in East African countries, the widely documented phenotypic groups include crested-head, frizzle, naked-neck, dwarf, tailless, bearded, rumples, normally feathered, and feathered-shank, among others (Figure 1). These genetic groups have been classified based on their morphological and phenotypic features, which are the results of genes with major phenotypic effects, known to contribute significantly to their adaptability and reproductive fitness in the tropical climatic environment [70]. Some of these genotypes are commonly distributed across different regions of these countries, whereas others are restricted in certain regions [54,66]. Normal feathered genotypes, crested head, feathered shank, and bearded genotypes are perceived to possess major genes with desirable effects for cold resistance. In Kenya, these genotypes are dominantly reported in high-altitude areas of Mount Kenya and the highlands East and West of the Rift Valley in Kenya [46,50,63], whereas in Tanzania, some of these rare genotypic groups were also reported by Guni et al. [54] in cold-weather regions of Jombe in Tanzania. This distribution could possibly be explained by the fact that these genotypes have more developed feathers covering their whole body, an attribute that insulates them from cold weather [70]. On the other hand, the naked-neck, frizzle, dwarf, and rumples genotypes are known to possess heat resistance genes, and for that reason, have been exceedingly reported in warm, humid, and hot climatic environments. Again, in Kenya, these genotypes were mostly reported in the Western and Coastal regions and in the Eastern and Northern lowland regions, all characterized by hot weather [50,63]. Similarly, in Tanzania, frizzle feathered genotypes are common in the Dodoma region and semi-arid regions of the country (author’s personal observation). Information on the distribution of these genotypes with respect to ecological zones in Uganda is still limited, although a similar observation has been reported elsewhere in Ethiopia [71]. In addition to their adaptive traits, genotypes with naked-neck and frizzle genes have been associated with increased growth rates, superior body weights, better feed conversion, higher egg production, as well as disease tolerance in tropical environments [48,63]. Furthermore, while the crested-head genotype is considered a superior egg producer in very cold environments, the bearded and feathered-shank genotypes have been elsewhere reported by Fayeye et al. [72] in Nigeria to have increased body weight and potential for egg mass production. Similarly, individuals’ genotypes with dwarf genes have been associated with better reproductive capacity (mothering ability), increased feed efficiency, and increased egg production in hot environments [48,73,74]. Despite the increasing evidence that some of these valuable genetic resources are in danger of extinction, they have not been fully exploited or utilized, nor conserved for present and future use.

### 6.3. Indigenous Chicken Ecotypes in East Africa (Agro-Ecological Zone Characterization)

As stated earlier, IC in some African countries have been characterized based on their geographical location and are here referred to as ecotypes. Thus, ecotypes are a group of IC found in one ecological zone or area as distinguished from another and are anticipated to possess a unique set of genes with special utility in the respective ecological area [70]. The names of the ecotypes are normally derived from the ecological zones of their location, and in some cases, regional names have been used [47,75]. Such distinct ecotypes found in distinct ecological zones have been reported in several countries, including Tanzania [52], Kenya [47], and Ethiopia [76].

In Tanzania, to date, nearly ten IC ecotypes from different ecological zones have been reported [31,52,53,77]. However, considering the enormous land expanse of Tanzania, coupled with the existence of diverse ecological zones, there is reason to suspect that IC have not been conclusively characterized [31,54]. The widely documented Tanzania IC ecotypes include Ching’weke (very short and compact ecotype, from Morogoro region), Mbeya ecotype (medium-sized ecotype, from high altitude region of Mbeya region), Pemba and Uguja (small-sized game birds from Pemba and Unguja Islands, respectively), N’zenzegere (medium-sized with frizzle plumage), Tanga (medium-sized birds from the coastal region of Tanga), Singamagazi (from Shinyanga region), and Kuchi and Horasi (heavy sized birds from Mwanza region). Despite the well-documented phenotypic variability that exists among these Tanzanian IC ecotypes [53,65], molecular characterization based on 29 microsatellites concluded that the five Tanzanian IC ecotypes involved in the study formed three distinct genetic groups related mainly to geographical distribution [78]. In this study, Ching’wekwe and Morogoro ecotypes from the Eastern and Central Zones of Tanzania’s mainland emerged into one distinct cluster, Unguja and Pemba Island game birds formed another distinct cluster, whereas the Kuchi ecotype forms a separate group (Figure 1). In recent years, the study by Mushi et al. [79] using 600 K SNP with three Tanzanian chicken ecotypes (Morogoro-medium, Chingwekwe, and Kuchi) reveals almost comparative findings, namely that Ching’wekwe and Morogoro-medium forms one genotype group, while Kuchi stands as a separate population. These related findings are, however, contrary to the baseline report by Msoffe et al. [75], who initially grouped Tanzanian IC ecotypes into nine separate genetic groups. However, it was worth noting that the reference study by Msoffe et al. [75], was based on few microsatellite markers, with only one of the 20 microsatellite markers present in the recommended list of markers proposed by FAO for chicken biodiversity studies [78]. Other studies have reported the genetic diversity of the IC in Tanzania [31]. In Kenya, indigenous ecotypes have also been studied in different regions of the country [80], and the ecotypes studied, such as Bondo, Bomet, Narok, Kakamega, West Pokot, Lamu, and Taita-Taveta, were named based on the regions of their location. Despite these geographically distributed IC groups, a molecular characterization study by Mwacharo et al. [81] using 30 microsatellite markers concluded that Kenyan IC could be grouped into four genetic groups, namely Coastal, Central, Western, and Northern Kenya. Later in 2013, the same authors using 30 autosomal microsatellite markers on genomic DNA identified two major gene pools, namely the Eastern and Western Kenya groups. In tandem with that study, Ngeno et al. [82] reported that Kenyan IC belongs to two to three genetically distinct groups depending on different clustering systems. MHC-linked markers divided IC into three mixed clusters composed of individuals from the different ecotypes, whereas non-MHC markers grouped IC into two groups (LM and others). Two main population clusters indicated are Lamu (one cluster) and populations from Kakamega, West Pokot, Turkana, Bomet, Narok, and Siaya as a second cluster, whereas an extra group (third cluster) was from Taita-Taveta. It is worth noting that although the two studies reported equal numbers of gene pools, the sampling sites were different.

## 7. Chicken Rearing, Production System in East Africa

Till recently, the poultry sector in East Africa consisted of three major production systems, namely [24,39,83]:

1. Commercial production system, i.e., commercial farming practices that are relatively modernized, with layers and broilers that are highly productive.
2. Semi-intensive production system, i.e., an intermediate system mainly with dual-purpose breeds with an average of 150 eggs per year and some attention to biosecurity.
3. Traditional, village, or free-ranging production system, i.e., backyard rearing of IC with an average of 50 eggs per year, 1.5 kg mature body weight, and limited attention to health care.

Nevertheless, according to the FAO definition, poultry production systems can be grouped into four main sectors (Table 5). Sectors 1 and 2 represent the large-scale commercial production system, which is relatively modernized. Sector 3 comprises the more intermediate small-scale commercial farms with dual-purpose birds, whereas sector 4 represents the backyard and scavenging system, which is largely based on indigenous birds [83]. Even if the ratio of the production systems (sectors) may differ from one country to another, still the village production system (Sector 4) accounts for a larger proportion and hence contributes a higher volume of meat and eggs in all three countries [24]. The commercial sector (Sector 1 companies) is led by a few large integrations, such as Kenchic in Kenya, Ugachick in Uganda, and Interchick, Kibo and Silvaland in Tanzania [24]. They supply day-old chicks and sometimes feed and other inputs to both large- and small-scale producers of broilers and layers. In Tanzania, there are very few producers in Sector 1, a few more in Sectors 2 and 3, but the overwhelming majority belongs in Sector 4 [83]. Nevertheless, due to the strongly growing demand for poultry products, the contribution of a more commercially oriented production system is growing in all African countries and is even expected to grow more in the coming decades with the growing population and urbanization, and rising income [24,40]. 

In the past few years, improved backyard production system (medium input–medium output) has become increasingly popular in East Africa, following the increased import of different dual-purpose breeds of chickens that are more productive than IC and less delicate than industrial breeds in developing market production environments [24,84]. The import of these breeds has become an emerging trend in the countries and has remained a major area of focus of numerous developmental projects and donors, including the Bill and Melinda Gates Foundation, as a tool for poverty reduction [85]. These birds have proved to offer good opportunities for poorer smallholders to gradually grow from an extensive backyard production system to a more market-oriented production system [85,86]. In Uganda, for example, Kuroiler, a dual-purpose breed from India known for laying up to 200 eggs a year [86], was first introduced by the National Animal Genetic Resource Centre (NAGRC) in 2011 for a trial study [87]. According to a recent report by Uganda’s Ministry of Agriculture, Animal Industry, and Fisheries, by the end of 2020, the government targeted the breeding and production of at least one billion poultry birds. Currently on the ground, the demand for Kuroiler chicken is high in Uganda likewise in Kenya, a neighboring country, whose first Kuroiler chicken import came from Uganda. Apart from Kuroier, other commercial breeding companies have started producing other dual-purpose hybrids in Kenya and other East African countries as well. Such breeding companies include Kenchic, producing the Kenbro, and Kukuchick, with the Rainbow Rooster, in Kenya, both with an estimated production capacity of over 1 million dual-purpose hybrids per year in commercial laying facilities. In 2014, Tanzania, Ethiopia, and Nigeria were lucky to host an Africa Chicken Genetic Gain project (ACGG) hosted by ILRI and partners, including the Bill and Melinda Gates Foundation. ACGG is part of the wider ‘Live Gene initiative’, which tests and makes available high-producing, farmer-preferred genotypes, both indigenous and imported, that are likely to increase smallholder chicken productivity in Africa [85]. In Tanzania, the project was implemented in five regions, and several improved dual-purpose breeds (Black Australorp, Kuroiler, and Sasso) were tested and disseminated. Further evaluation studies showed that the Kuroiler and Sasso breeds had been accepted by the farmers in Tanzania, owing to their higher meat and egg yields relative to indigenous birds in the scavenging production system. Moreover, the entrepreneurship opportunities that existed along the value chain (mini hatcheries, brooding houses, and crossbreeding activities) have also continued to attract the attention of many youths, men, and women, hastening their adoption process. In recent days a researcher from Tanzania who tried to analyze the adoption trend of these breeds argued that if extension efforts to facilitate the availability of these breeds are maintained, adoption of these improved breeds may increase up to 59% in the next 8 years in selected areas [88].

## 8. Past, Present, and Future Genetic Improvement Programs of Indigenous in East Africa

It is undeniable that rural chicken production plays a vital role in the daily lives of poor farmers in most developing countries. Farmers prefer keeping IC over exotic breeds because of their small cost of production, scavenging capacity, and adaptability to harsh environmental conditions, and many other reasons [89]. However, despite the increasing evidence to demonstrate the role of rural poultry farming in the lives of resource-challenged families, their overall contribution is yet very low, notwithstanding their huge number, estimated to be over 80% of the total poultry population in most tropical countries [89,90]. Comparative performance studies have indicated that IC attained their mature body size of about 1 kg at the age of about 16–20 weeks, compared to commercial broilers, which reach a market weight of approximately 2 kg and above, in less than 8 weeks, whereas crossbreeds attain the same weight at 12 weeks under intensive management [65,91,92]. Yet again, IC attains their sexual maturity 60 days later and achieves up to less than 60% of the annual egg production compared to commercial layers [92,93]. This level of productivity for IC is very low; therefore IC are not suitable for poor farmers to produce with the aim of improving their livelihood and meeting the currently rapidly increasing demand [89,94]. On the contrary, the use of exotic breeds in tropical countries requires more inputs and high management skills far beyond the ability of ordinary farmers [91,95,96]. Consequently, previous research has indicated the existence of a potential for the existing productivity gap to be reduced through the implementation of different interventions existing in the village poultry production system, for example, provision of vaccination, improved feeding, clean water, and provision of improved housing [62,93]. However, this option is often less attractive to farmers because such high expenditure is considered too risky, considering the low productivity of the IC given the production system [93]. Since IC genetic resources can make the best use of their actual environment, genetic improvement of IC in terms of productivity would contribute a great deal in improving village poultry production. Since the 1960s, several attempts aiming at addressing the inherently low productivity of IC have been made in these countries with limited success, the latest being the introduction of market-oriented dual-purpose chicken breeds, including Kuroiler, a dual-purpose breed from India. Some of these attempts had minimal success due to several reasons, including lack of a holistic approach in solving the constraints and the dissemination of inappropriate technologies given the production circumstances. Other common attempts of genetic improvement in the past include:

1. Importation of pure temperate exotic breeds, where breeds such as White and Brown Leghorns and Rhode Island Reds were imported over the years from the 1960s;
2. Crossbreeding/upgrading program of unselected IC chicken breeds with imported exotic breeds, which involved a cockerel distribution scheme and change programs;
3. Limited selection within IC for improved performance.

Even though genetic improvement within breed selection remained the most viable option considering its contribution to sustainability by conserving the local gene pool, the components of reproduction under low-input scavenging production remain very complex, making selection exceedingly difficult. Indeed, there are several cases where performance has been improved through this approach, but they are few, and the gains have been modest [92]. A researcher from Tanzania concluded that selection for dual-purpose characteristics within individual indigenous populations is both time-consuming and costly [97]. Consequently, due to the shortcomings mentioned, crossbreeding followed by selection in the composite population was accepted in principle and practice as a shortcut for the genetic improvement of indigenous livestock [93]. In many regions, this program involved the crossbreeding of unselected IC to different levels of exotic blood in attempts to provide birds that are tolerant to local conditions while also capable of reasonable performance. Nearly all crossbreeding programs were partially successful in terms of increased performance in crossbred chicken, but again they were not sustainable in low-input systems, which consequently led to their termination. The common factors recognized were: incompatibility of genotypes with farmers’ breeding objectives, failure of controlling breeding to maintain heterosis given the village environment, lack of a sustainable breeding program to supply breeding cocks, high cost of maintaining breeding cocks, and it also occurred that the acquired exotic cocks were not as lively and active as IC under village conditions [30,98]. Increasing the level of exotic blood also resulted in the loss of brooding behavior and the ability to evade predation by crossbred birds, both traits being of considerable economic value in village systems [30,48]. Furthermore, the improved crossbred birds were often reported to require additional management to achieve their full genetic potential for production, and thus smallholder farmers were not prepared to adopt them owing to economic, social, or cultural reasons.

## 9. Future Management and Breeding Plan for Developing Chicken Breeding in East Africa

Livestock genetic diversity is a critical factor in ensuring productivity and adaptability of livestock breeds, facilitating resilience to climate change and long-term food security all around the world [99,100]. It has been extensively demonstrated in most tropical countries that the smallholder production system represents a unique reservoir of genetic resources [70,99]. Several indigenous chicken breeds have been reported to possess both superior levels of genetic variation relative to commercial breeds and unique phenotypic traits signifying valuable local adaptations. Many of these breeds, including IC, are adapted to harsh environmental conditions, poor nutritional regimes, the ravages of climate, and diseases compared to exotic breeds, which improve their resilience in the challenging and changing ecological terrains of Africa [100]. It has been proposed that breed improvement and subsequent proper utilization of IC genotypes require comprehensive characterization, including breeding practice [101]. Nevertheless, the IC of Africa have for a long time remained poorly characterized, and therefore rational decisions for their improvement are limited. Furthermore, there is also widespread concern in developing countries that as a result of the replacement of IC ecotypes with high-producing breeds, indiscriminate crossbreeding, high intake and offtake rate of IC promoted by their prolific nature, economic drivers, urbanization, weak policies on the protection of animal genetic resources, changes in the production system, and many other reasons, the world continues to lose valuable and irreplaceable poultry genetic material. In the recent past, for instance, owing to the currently emerging intermediate production system, indiscriminate crossbreeding practices of IC with the lately introduced dual-purpose breeds in the endeavor to upgrade the IC whose productivity is deemed to be low is increasingly becoming popular among East African rural farmers. Indeed, in principle, indiscriminate crossbreeding activities are discouraged, however owing to the complexity of the traditional chicken production system where breeding is completely uncontrolled, replacement stocks are produced through natural incubation using broody hens while systematic records are not kept, indiscriminate crossbreeding can hardly be controlled. Worse still, in some developing countries, such as Tanzania, there is no clear legislation or breeding policy that outlaws indiscriminate crossbreeding practices, and, as a consequence, these activities are left to be done chaotically and against the Global Plan of Action for Animal Genetic Resources. On the other hand, concerns about a loss in genetic variability in commercial poultry strains have also been raised in the past decades following dramatic global reductions in the number of commercial poultry breeders and the number of populations under selection that could place the industry in danger in the event of a major disease outbreak involving new virus strains, particularly in the face of global climate change [102]. Thus, it would be more than a loss for the poultry industry in general if IC breeds, which are recognized as the reservoir of genomes and major genes of economic importance for the future development of commercial poultry breeds, are lost for the sake of short-term benefits. Furthermore, considering how smallholder poultry production affects the livelihoods of the majority of the rural population in most developing countries, as well as their nutritional role that cannot easily be substituted by other kinds of animal production, the future planning of a genetic improvement program, should therefore focus on understanding natural genetic variation in indigenous livestock breeds, and then finding the balance between the competing needs of genetic improvement and genetic diversity [100]. In the recent past, the applications of biotechnology in the areas of animal genetics and breeding have opened the door for animal breeders to overcome the above limitation. Marker identification and use should be therefore expected to enhance prospects of breeding for the productive and adaptive traits of IC in East Africa and beyond [100,103]. 

The link between individual genes and productivity in the smallholder poultry production system has been successfully researched in various tropical countries in the Middle East, Africa, Asia, and elsewhere [104,105]. Among the first genes to be reported under selection in domestic chicken is the thyroid-stimulating hormone receptor (TSHR) locus, which was initially linked to the improvement of egg productivity [106], and from that point on, different studies have detected similar selection signatures at the TSHR locus in several IC populations found in different agro-ecologies [104]. Evidence has also shown that IC populations have revealed robust results to selection for resistance to food and water scarcity, diseases, parasitic infection, as well as hot climatic conditions [104,105,107]. Moreover, morphological and phenotypic traits, such as the naked neck, frizzled feathers, large combs, large wattles, and long legs, have been studied, and the results have indicated that some of these traits are related to tropical adaptability and productivity traits [101]. In practice, naked neck and frizzling genes were utilized for the improvement of tropical adaptability in high-producing broiler and layer populations in Israel and India. Furthermore, it has been reported that the BL-β II gene for disease resistance has been cloned from the Aseel breed of Indian chicken [30]. Nevertheless, it is considered highly probable that these traits are not encoded by single major genes but are the result of the interaction of multiple genes [101], thus it could also be highly desirable to be incorporated into the development of high-performance IC for the tropics. For instance, recent evidence revealed some genomic regions under positive selection which are associated with water scarcity, scavenging challenges, feeding behavior, and altitude-induced stresses from a population of 245 Ethiopian IC selected from 34 different agro-ecological zones [108]. Despite the increased awareness about the importance of indigenous animal breeds, which includes their long history of adaptation to extreme habitats, little effort has been done to harness the genetic potential of African IC ecotypes in general. Until recently, only a little research and development activity had been directed towards phenotypic and genetic characterization of IC populations in Africa, and, wherever applicable, weak genomic tools, such as microsatellite markers that produce limited information, were often applied. It is therefore likely that the genetic potential of some African IC populations has been underestimated in previous studies.

## 10. Limitation, Conclusions, and Future Research Directions 

This study has a number of potential limitations that may influence or limit the scope of the study in one way or the other. Foremost, there is a notifiable research gap in the literature with respect to the genetic aspects of indigenous chickens in the studied countries and Africa in general. As highlighted earlier in the main text, several indigenous chickens have yet not been fully classified into standard breeds and instead are habitually described, named, and grouped according to geographical location or phenotypic characteristics. As a result, previous investigation studies on either productive, reproduction, nutritional requirements, and adaptability potential of IC have mostly ended up with inconclusive and contradictory results. Practically, on the ground and due to limited previous research studies on indigenous breeds, this review did not, for the most part, gather enough literature addressing the characterization and distribution of IC breeds of Uganda compared to the other two countries. Similarly, most of the available food security assessment reports in the studied countries address the issue of food security in relation to the availability of cereal crops (grains) and not access to livestock. Thus, by referring to a broader concept of food security, this observation is misleading, particularly among pastoralists and agropastoral communities whose households largely (more than fifty percent) depend on livestock for their livelihood.

Nevertheless, on the basis of the important findings of the study, this discussion concludes that the need for genetic improvement and conservation of IC chicken population genetics is a factor that needs urgent attention considering how these poultry species strongly affect the livelihoods of the majority of rural people in most developing countries. Furthermore, since the livestock sector is likely to be strongly affected by climate change, the adaptability features of IC to low input environments across diverse agro-ecological conditions also provides a unique genetic resource that needs to address the global challenges of food security, as well as the opportunity to understand the mechanism behind their adaptation to climate change. Thus, the anticipated areas of research that will continue to attract the attention of many researchers in the coming years embrace the need to understanding how climate change may have generated new adaptive responses across the chicken genome, the need to understand genetic and non-genetic factors underlying the tolerance and disease resistance potential of IC found in different ecological zones, the extent to which adaptation of IC to the local environment has continued to shape their phenotypic future, behavior and productivity, and many other comparable topics. All these interesting research questions require insight from both genetic and epigenetic mechanisms and, for that reason, the establishment of a positive enabling environment for molecular technological research and funding support for local genetic resource conservation programs that could have positive effects on the sustainable growth of the poultry industry in East Africa and beyond, and hence broader global food security goals.

## Figures and Tables

**Table 1:** Global Hunger Index and nutritional indicators for Kenya, Uganda, and Tanzania in 2020.

Country	Nutritional Indicators	Years			
		2000	2006	2012	2020
Kenya	Global Hunger Index	37.4	31.4	23.2	23.7
	Proportion of the undernourished population (%)	32.4	26.3	23.2	23.0
	Prevalence of wasting in children under 5 years (%)	7.4	6.9	4.2	4.9
	Prevalence of stunting in children under 5 years (%)	40.8	40.3	26.2	31.3
	Under-five mortality rate (%)	10.6	7.4	5.2	4.1
Tanzania	Global Hunger Index	40.8	33.6	30	25
	Proportion of the undernourished population (%)	33.1	30.3	29.1	25
	Prevalence of wasting in children under 5 years (%)	5.6	3.5	5.3	3.5
	Prevalence of stunting in children under 5 years (%)	48.3	44.4	36.2	31.8
	Under-five mortality rate (%)	13	8.9	6.6	5.3
Uganda	Global Hunger Index	-	-	-	30.6
	Proportion of the undernourished population (%)	-	-	-	-
	Prevalence of wasting in children under 5 years (%)	5	6.2	4.2	3.5
	Prevalence of stunting in children under 5 years (%)	44.9	38.4	33.7	28.9
	Under-five mortality rate (%)	14.8	10.2	6.7	4.6

Source [15] Note: Data underlying the calculation of child stunting and child wasting are from 1998–2002 (2000), 2004–2008 (2006), 2010–2014 (2012), and 2015–2019 (2020). Data for undernourishment are from 2000–2002 (2000), 2005–2007 (2006), 2011–2013 (2012), and 2017–2019 (2020).

**Table 2:** Summary of poultry population and annual egg production by breed in Uganda from 2014–2018.

Year	Chicken Population (Numbers)			Egg Production (Millions)			Milk Production (Litres)
	Indigenous	Exotic	Total	Indigenous	Exotic	Total	
2014	39,206	914	40,120	685.5	171.4	856.8	1550
2015	40,382	941	41,323	706.0	176.5	882.5	1569
2016	40,597	991	41,588	706.1	176.5	882.6	1634
2017	41,726	918	41,726	725.7	181.4	907.1	1614
2018	42,885	6014	48,901	744.5	186.2	930.7	2040

Source [37].

**Table 3:** Five years national livestock population and production trend in Tanzania from 2015 to 2020.

Livestock Production Estimates	Financial Years				
	2016	2017	2018	2019	2020
Milk production (000′ liters)	2,127,267	2,087,000	2,400,134	2,678,461	3,002,555
Egg production (000′)	4,353,182	2,758,000	3,156,692	3,575,621	4,051,179
Total meat production (all kind) in metric tons	636,559	621,697	679,992	690,629	701,679.1
Poultry meat (metric tons)	104,292	63,597	78,110	79,332	80,601.3
Poultry as % of total meat production	16.4	10.2	11.5	11.5	11.5

Source: [41].

**Table 4:** Mean production and reproduction performance of the indigenous chicken population.

Parameters	Intensive Management	Extensive Management	References
Weight of adult cocks (kg)	2.21	1.8	[66]
Weight of adult hens (kg)	1.7	1.3	[66]
Weight at 20 weeks (kg)	1.6	1.12	[65]
Bodyweight at 8 weeks (g)	0.45	0.31	[65]
	0.44	-	[66]
Age at first egg (months)	166	224	[67]
	168–173	-	[65]
	-	7.3	[65]
	-	7.48	[54]
	-	6.2	[46]
Clutch size/hen	-	13.7	[54]
	-	11.8	[64]
	-	14.1	[45]
Clutch interval (months)	-	2-3	[68]
	-	2.7	[45]
Number of clutches per year	-	3.3	[54]
	-	2.68	[64]
	-	2.2	[45]
	-	3-4	[68]
Estimated eggs/year	-	45	[54]
	-	31.6	[64]
		87.2	[45]
	-	81.5	[69]
	-	90.0	[68]
Chick mortality rate (%)	-	30.2	[54]
	-	28.95	[65]
	-	39.1	[69]

**Table 5:** FAO classification of poultry production systems.

Sector Designation	Production System	Characteristics
1	Industrial integrated	High level of biosecurity; birds/products marketed commercially (farms that are part of an integrated broiler-production enterprise with clearly defined and implemented standard operating procedures for biosecurity).
2	Commercial 1	Moderate to high biosecurity; birds/products are usually marketed commercialy. (farms with birds kept indoors continuously; strictly preventing contact with other poultry or wildlife)
3	Commercial 2	Low to minimal biosecurity; birds/products sold in live bird markets eg. a caged layer farm with birds in open sheds, a farm with poultry spending time outside the shed, a farm producing chickens and waterfowl.
4	Village backyard production	Minimal to no biosecurity; the birds’ products are consumed locally.

## Data Availability

Not applicable.
